# Factors Related to Smoking Habits of Male Adolescents

**DOI:** 10.1186/1617-9625-2-13

**Published:** 2004-09-15

**Authors:** Nyi Nyi Naing, Zulkifli Ahmad, Razlan Musa, Farique Rizal Abdul Hamid, Haslan Ghazali, Mohd Hilmi Abu Bakar

**Affiliations:** 1 Department of Community Medicine, School of Medical Sciences Universiti Sains Malaysia, 16150 Kubang Kerian, Kelantan, Malaysia

## Abstract

A cross-sectional study was conducted to identify the factors related to smoking habits of adolescents among secondary school boys in Kelantan state, Malaysia. A total of 451 upper secondary male students from day, boarding and vocational schools were investigated using a structured questionnaire. Cluster sampling was applied to achieve the required sample size. The significant findings included: 1) the highest prevalence of smoking was found among schoolboys from the vocational school; 2) mean duration of smoking was 2.5 years; 3) there were significant associations between smoking status and parents' smoking history, academic performance, perception of the health hazards of smoking, and type of school attended. Peer influence was the major reason students gave for taking up the habit. Religion was most often indicated by non-smokers as their reason for not smoking. Approximately 3/5 of the smokers had considered quitting and 45% of them had tried at least once to stop smoking. Mass media was indicated as the best information source for the students to acquire knowledge about negative aspects of the smoking habit. The authors believe an epidemic of tobacco use is imminent if drastic action is not taken, and recommend that anti-smoking campaigns with an emphasis on the religious aspect should start as early as in primary school. Intervention programs to encourage behavior modification of adolescents are also recommended.

## Introduction

Smoking is the single most important preventable cause of death [1]. The secondary school age is a critical period in the formation of the smoking habit. Most smokers start smoking during their adolescence or early adult years. The earlier they start to smoke, the more likely they are to become regular smokers [2,3]. Those concerned about the health, welfare and education of young people should be anxious to find ways to prevent them from taking up this habit.

In Malaysia, with the improvement of socioeconomic status and the standard of health care, the incidence of communicable diseases has declined significantly, but other health problems are emerging. An example of the diseases related to smoking is coronary artery disease, which now is the main cause of death in hospitals in peninsular Malaysia [4].

Adolescent and teenage smoking have been studied widely, and it has been found in developed countries that nearly one-half of school students who have reached the age of 18 have already established the habit of smoking with some degree of regularity, and it is a rather unrealistic hope on the part of adults to expect that children will abstain until reaching the adult approved age of decision [5].

Smoking is a major problem among youth in Malaysia. In a recent survey by the Ministry of Youth and Sports on negative behaviors among 5,860 adolescents, 80% indicated that they had ever experienced smoking [6]. Schooling is the major activity of most children between the ages of 7 and 17 years and school is the place where most of them socialize outside their home environment for the first time. A school is the place where much knowledge is obtained, attitudes are formed and sometimes habits are chosen. Studies have demonstrated that the secondary school age is a critical period in the formation of the smoking habit [7]. Experimenting with cigarettes often begins during childhood or early adolescence and there is usually a period of about 1.5 to 2 years between initiation of smoking and establishment of the smoking habit [8,9].

Schooling is compulsory in Malaysia. A child enters school at the age of 7 and attends primary school for six years, after which he enters lower secondary school (Form I–III). At the end of Form III there is a qualifying examination, and only students who pass this examination proceed to upper secondary school (Form IV–V). Those who fail repeat Form III, go to vocational or private schools, or drop out of school [10].

A number of factors influence an individual to start smoking. Lack of awareness and knowledge have been reported as contributing factors based on studies in the past. This study aimed to highlight environmental, religious and other factors influencing smoking in male adolescents in secondary schools.

## Methods

A cross-sectional study was conducted during January through June 2001. Applying the cluster sampling method, 451 Form IV and V (upper secondary) male students from three different schools (day, boarding and vocational) were included in the study. The reason for selecting different schools was to examine smoking habits in relation to the nature of the schools attended by the students. Only male adolescents were included in the study. The number of participants from each school were: 150 from day school (33.26%), 150 from boarding school (33.26%) and 151 from vocational school (33.48%). Since the population of Kelantan state is 90% Islamic, all study participants were Malay Muslim schoolboys. A structured questionnaire was distributed among them for self-administration with prior explanation. Subjects were asked to indicate their reasons for smoking or non-smoking based on factors such as religion, parental control, parental smoking status, peer influence, feeling of maturity, enjoyment, monetary and other factors. The validity and reliability of the questionnaire was tested earlier based on pre-test results, using statistical software. Smoking status was self-reported, and was not verified by any biochemical measures. The Pearson Chi-square test was applied to determine the statistical significance of association at 5% level of significance. The data was analyzed using SPSS software, version 10.0.

Definition of terms used were:

Tried/smoked before: one who has only tried smoking and is not a smoker now, or one who has previously smoked but is currently not a smoker;

Never tried: one who has never tried smoking in his life;

Current smoker: one who smokes currently, regardless of frequency and amount smoked.

## Results

Mean age of subjects was 16.46 years. Current smokers comprised 35.92% of 451 male students surveyed. Vocational school students had the highest proportion of smokers. Among non-smokers, 187 students had smoked some time in their lives. Mean duration of smoking was 2.49 years. Approximately two-thirds of the smokers started the smoking habit before the age of 15. A total of nearly 21% of the students smoked daily for more than three years (Table 1).

**Table 1:** Duration of smoking by type of smokers

Duration	Type of Smoker (years)		Total
		
	Regular n (%)	Occasional n (%)	
< 1	19 (20.88)	35 (49.30)	54 (33.33)
1–2	36 (39.5)	22 (30.98)	58 (35.81)
3–4	19 (20.88)	9 (12.68)	28 (17.28)
5 >	17 (18.68)	5 (7.04)	22 (13.58)
Total	91 (100.00)	71 (100.00)	162 (100.00)
Mean (years)	3.26	2.51	2.49
SD (years)	1.45	1.40	1.47

**Figure 1:**
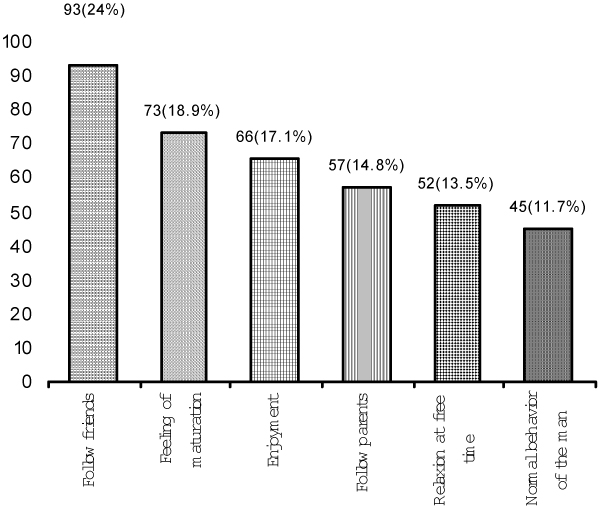
**Reasons for smoking, among smokers**.

**Figure 2:**
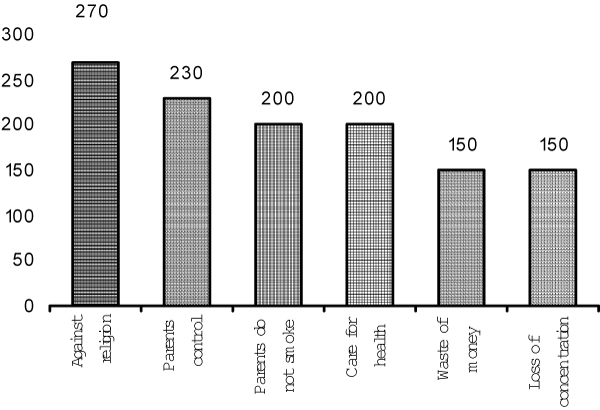
**Reasons for not smoking, among non-smokers**.

A significant proportion of the smokers (41.98%) smoked more than 10 cigarettes per day. There was a significant association between the smoking habit of the fathers and that of the students (p < 0.01) (Table 2). Reasons most often given for smoking were: following friends, feeling of maturation, enjoyment, following parents, relaxation in free time, and feeling that smoking is the normal behavior of a man (Figure 1). Non-smokers most often cited religion, parents' influence, health protection, and financial reasons as factors preventing them from smoking (Figure 2).

**Table 2:** Association between smoking status of students and selected variables

	**Smoking Status of Students**			
			
**Variable**	**Smoker n (%)**	**Non Smoker n (%)**	**Test Statistic**	p-value*
**Smoking status of father**				
Smoker	71 (44.10)	90 (55.90)	7.28	0.007
Non – smoker	91 (31.38)	199 (68.62)		
**Knowledge on smoking as harmful to health**				
Agreed	122 (75.31)	257 (88.93)	16.89	< 0.001
Disagreed	21 (12.96)	11 (3.81)		
No opinion	19 (11.73)	21 (7.26)		
**Level of academic performance**				
Good	44 (25.14)	131 (74.86)	14.43	< 0.001
Poor	118 (42.75)	158 (57.25)		
**Type of school**				
Vocational school	84 (55.6)	67 (44.4)	48.10	< 0.001
Day school	52 (34.7)	98 (65.3)		
Boarding school	26 (17.3)	124 (82.7)		

*Chi-square test is applied

**Figure 3:**
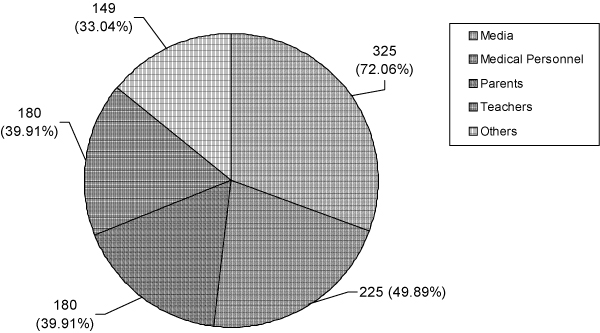
**Sources of knowledge about the hazards of smoking**.

The perception of the health hazards of smoking was significantly different between smokers and non-smokers (p < 0.001) (Table 2). Smokers had relatively poor academic performance compared to non-smokers (p < 0.001) (Table 2). The proportions of smokers in the three schools were significantly different (p < 0.001). The vocational school had the highest number of smokers compared to the other two (Table 2). About 60% of smokers had thought of quitting smoking and of these nearly 45% had tried at least once to quit (Table 3). Mass media was cited as the best source of information about smoking hazards, followed by medical personnel (Figure 3). There was no significant association between source of knowledge and smoking status of the students.

**Table 3:** Wish to stop and attempted quitting of smoking, by type of smokers

	**Type of Smoker**		
		
**Factor**	**Regular**	**Occasional n (%)**	**Total**
*Wish to stop smoking			
Yes	51 (56.05)	46 (64.79)	97 (59.88)
No	16 (17.58)	14 (19.72)	30 (18.52)
Uncertain	24 (26.37)	11 (15.49)	35 (21.60)
Total	91 (100.00)	71 (100.00)	162 (100.00)

**Attempted quitting			
Tried	32 (35.16)	40 (56.34)	72 (44.44)
Did not try	59 (64.84)	31 (43.66)	90 (55.56)
Total	91 (100.00)	71 (100.00)	162 (100.00)

*P *< 0.05 **P < 0.001

*x^2 ^= 2.79 **x^2 ^= 7.24

## Discussion

In Kelantan, where the community is relatively conservative, the prevalence of smoking of 35.9% among male adolescents reported by this study was high. The prevalence of male adolescent smoking in Malaysia was reported as 30.7% by the National Health Morbidity survey conducted nationwide in 1996 [11]. Our study has shown an even higher prevalence than the national level. The authors believe this may be because of the homogeneity of study participants, who were from only the Malay ethnic group. The National Health Morbidity Survey indicated that adolescent smoking was highest in Bumiputras [11]. The races included under Bumiputra status are Malays and other local tribes. The Non-Bumiputras category includes Chinese and Indians who migrated to Malaysia some decades ago. While the results of our study may not be generalizable to the entire population, this study supports the fact that smoking is a serious problem among the majority ethnic group in the Malaysian population. Among a few locally conducted studies, Thambypillai in 1985 found a prevalence of 17.0% among secondary school boys in urban school in Kuala Lumpur, Malaysia [9]. In Saudi Arabia, the prevalence of smoking among secondary school boys was only 17% while in China, among middle school students, it was only 2.24% [12,13].

Among the schools where the study was carried out, the highest smoking prevalence was found in vocational school, as expected. Those who entered vocational schools had relatively poorer academic performance than those who went to the other two types of schools. This is no doubt due to the fact that those who fail the qualifying examinations in Form III have to leave school or join vocational or private schools. This study points out that the likelihood of being a smoker increases among academically poorly performing students. Thambypillai in 1985 reported a similar finding of association between high smoking prevalence and poor academic performance [10].

Our study found that peer influence is the major reason for initiation of smoking; this is similar to findings of other studies [14]. Peer influence was found to be a strong predictor of smoking initiation in almost all studies that included these measures. Similar findings have been documented by the Surgeon General of the United States [2]. Two types of peer pressure (i.e., having close friends who smoked, and having close friends who encouraged the student to smoke) were among the strongest risk factors for both regular and occasional smoking. Similar results were found in reports on junior and senior high school students in China [15,16]. Studies from Japan [17] and Spain [18] have shown that smoking rates of school students are strongly related to having friends who smoke. This suggests that pupils should be advised to avoid accepting smokers as friends, as the effects are not limited to the odor of tobacco and hazards of passive smoking, but may include pressure to take up this habit.

This study found religion was the strongest reason among non-smokers for not smoking. A similar finding has been shown in earlier studies in Saudi Arabia of schoolboys [19], medical students [20] and university students [21]. Smoking has not been declared as "forbidden" from the Islamic religious point of view in Malaysia. However, Malaysia has experienced rapid economic progress in recent years and as the nation has opened to advertising, marketing and imports by international tobacco corporations, smoking rates among teenagers and adolescents have increased [22]. We recommend that religious education including a religious perspective on the smoking issue should be more emphasized in school curriculum and anti-smoking campaigns, since it is potentially an effective way to educate students especially at the secondary school age. While our study found mass media were the source of information on smoking more than medical personnel, parents, teachers and others, we point out that mass media can not replace face-to-face communication between a student and a doctor, a teacher or parents.

Parental smoking has great influence on the children with respect to taking up the smoking habit. There was a significant association between the students' smoking status and their fathers' smoking habits found in this study. The social learning theory of behavior [23] states that children are more likely to model their own behavior on actions of people they regard as worthy, similar to themselves, and models of their own sex. Two studies [24,25] reported that parental attitude in actively discouraging their children from smoking may be more powerful than parental behavior in shaping adolescent cigarette smoking behavior. Two other studies [26,27] reported that the majority of smokers began their habits in imitation of friends, co-workers or family members. This suggests that in order for campaigns against smoking in adolescents to be most effective, parents must not smoke in the presence of children. More importantly, the children must not be allowed to smoke in their parents' presence. The home environment should complement the school to discipline the students. The role model should be set at home.

Academic performance of the students had a highly significant association with the smoking status of the students. Smokers had marked poorer performance than the non-smokers. This finding supports the findings reported by University of Malaya in 1985 in which a higher prevalence of smoking was shown to be associated with poor academic performance [28]. We call on school authorities to conduct more regular exhibitions and talks, with the help of health personnel, to educate the students about the negative impact of smoking on learning activities. We also recommend that sports and other co-curriculum activities should be encouraged more, in order to provide ways for students to be constructively occupied to prevent them from being attracted to unwanted vice.

There was a statistically significant difference in perception towards hazards of smoking between smokers and non-smokers shown by this study. Agreement on the harmfulness of smoking to health was higher among non-smoking students. A similar finding was shown by a study published in 1991 [29]. Even though smoking may not be regarded as a major discipline problem, the main adverse effects on health are well documented. Smoking is considered the main avoidable cause of death and the most important public health risk. The hazards of tobacco affect not only smokers, but also non-smokers who are exposed to cigarette smoke, "passive smokers." The earlier a person starts smoking, the more difficult it is to quit, the less likely it is that he will quit, [30] and the greater the risk of lung cancer [31] or death from coronary heart disease [32]. The chances of success in quitting decrease as age increases [33]. To develop and implement effective measures of smoking control, one must understand the reasons and risk factors for smoking initiation.

Information about health hazards is usually insufficient for change; studies in Indonesia and other countries in Asia confirmed the observation that knowledge of health risks does not prevent children from smoking [34]. In addition to demonstrating the hazards of smoking, the benefits of quitting should also be stressed. This study found that more than 50% of regular and occasional smokers expressed their wish to stop smoking. Nearly 45% of them attempted quitting, though a significantly higher proportion was found among occasional smokers. This shows that there was a desire to quit smoking despite the fact that it is difficult to stop the habit once it has become established.

While new and innovative approaches to smoking prevention and cessation are being sought, the addictive nature of cigarette smoking and the health advantages of stopping smoking should be given further emphasis in current prevention programs among adolescents. Community and school education programs should include sessions on quitting smoking since there are scientifically proven cessation methods available now. Efforts to prevent experimentation with smoking must also be given higher priority. Promising strategies may include a ban on cigarette advertising and higher tobacco taxes. There is considerable evidence that tobacco advertising and promotion encourage adolescents to smoke [35,36] and that increasing the price of cigarettes discourages young people from starting to smoke.

This study focused on smoking behavior on male adolescents. Schoolgirls were not included in the study because in the cultural setting chosen for the study smoking is considered primarily a problem of males, and because of the small sample size of the girls in the schools which were chosen to be included in the study. Also, an unpublished report by the state education department authorities indicated a very small proportion of the smokers among Muslim girls. However, the National Health Morbidity Survey conducted in 1996 reported that the prevalence of female adolescent smoking was as high as 4.8% [10]. This was an alarming reminder to parents in Malaysia that girls, as well as boys, are at risk to take up smoking. Other studies [10,26,37] conducted in Western countries show a higher percentage of smokers among boys and girls of the same age. Given the increasing influence of western culture in Asia and other parts of the world, we would recommend a study exploring smoking behavior among Malaysian female adolescents.

The magnitude of the smoking problem in adolescents is large enough to be considered a warning of an impending epidemic. Intervention programs that focus on behavior modification of adolescents should be carried out on a large scale. Multi-centered studies with a homogenous population would be appropriate to measure the effectiveness of intervention strategies.

## Competing interests

The authors declare that they have no competing interests.
